# Choroidal Thickness Analysis in Patients with Usher Syndrome Type 2 Using EDI OCT

**DOI:** 10.1155/2015/189140

**Published:** 2015-05-17

**Authors:** L. Colombo, B. Sala, G. Montesano, C. Pierrottet, S. De Cillà, P. Maltese, M. Bertelli, L. Rossetti

**Affiliations:** ^1^Department of Ophthalmology, San Paolo Hospital, University of Milan, 20142 Milan, Italy; ^2^Vita Salute San Raffaele University, 20132 Milan, Italy; ^3^Department of Ophthalmology, AO Maggiore della Carità, 28100 Novara, Italy; ^4^MAGI Human Medical Genetics Institute, 38068 Rovereto, Italy

## Abstract

To portray Usher Syndrome type 2, analyzing choroidal thickness and comparing data reported in published literature on RP and healthy subjects. *Methods*. 20 eyes of 10 patients with clinical signs and genetic diagnosis of Usher Syndrome type 2. Each patient underwent a complete ophthalmologic examination including Best Corrected Visual Acuity (BCVA), intraocular pressure (IOP), axial length (AL), automated visual field (VF), and EDI OCT. Both retinal and choroidal measures were measured. Statistical analysis was performed to correlate choroidal thickness with age, BCVA, IOP, AL, VF, and RT. Comparison with data about healthy people and nonsyndromic RP patients was performed. *Results*. Mean subfoveal choroidal thickness (SFCT) was 248.21 ± 79.88 microns. SFCT was statistically significant correlated with age (correlation coefficient −0.7248179, *p* < 0.01). No statistically significant correlation was found between SFCT and BCVA, IOP, AL, VF, and RT. SFCT was reduced if compared to healthy subjects (*p* < 0.01). No difference was found when compared to choroidal thickness from nonsyndromic RP patients (*p* = 0.2138). *Conclusions*. Our study demonstrated in vivo choroidal thickness reduction in patients with Usher Syndrome type 2. These data are important for the comprehension of mechanisms of disease and for the evaluation of therapeutic approaches.

## 1. Introduction

The choroid plays several important functions within the eye, providing metabolic support to retinal pigment epithelium (RPE) and outer retina, contributing to the blood vascularization of the preliminary portion of the optic nerve and absorbing excess of light entering the retina and RPE thanks to the presence of melanocytes.

The choroid cannot be excluded in the evaluation and understanding of the pathophysiological mechanisms responsible for diseases of the posterior segment.

Many experiments and clinical studies have shown both retinal and choroidal altered blood flow in Retinitis Pigmentosa (RP) [1–3]. Clinically, the retinal vessels reduction is a pathognomonic finding in RP and the value of retinal blood flow measurement is significantly reduced in RP patients compared to that in healthy subjects [2]. Similarly in patients with RP, the ocular pulse amplitude (OPA), an indirect measure of choroidal perfusion, was significantly reduced in the advanced stages of the disease [4]. However, the role that these perfusion abnormalities have in the pathogenesis of RP is still uncertain.

The recent development of the technique Enhanced Depth Imaging (EDI) [5, 6], which allows through the use of Spectral Domain OCT obtaining detailed images of the choroid, has given substantial impetus to the study of the physiopathological role of choroid such as physiological variation with age and implication in pathogenic mechanisms.

In RP, the analysis of choroidal thickness has a double importance; while it is an important additional factor for the understanding of the pathogenesis and course of the disease, it is an essential element in the evaluation of therapeutic perspectives, like retinal implants, gene therapy, and stem cells therapy.

Retinitis Pigmentosa is also a term that embraces a multitude of clinical conditions that have similar characteristics but differ for age of onset, course, and severity.

The aim of the study is to portray the most common syndromic form of RP, Usher Syndrome type 2, analyzing the thickness of the choroid in correlation with disease stage and comparing values reported in literature on nonsyndromic RP and healthy subjects.

## 2. Methods

All patients were recruited from the hereditary retinal diseases service at the University Eye Clinic of San Paolo Hospital in Milan between January and May 2014. They are part of a larger group of Usher Syndrome patients being evaluated at our clinic. Diagnosis of Usher Syndrome was confirmed by both clinical signs (characteristic bone spicule pigmentation, optic disc pallor, retinal vessel attenuation, visual field constriction, flat electroretinographic waves, photoreceptor atrophy shown by OCT, and bilateral sensorineural hearing loss) and results of genetic analysis.

We considered for the present study patients with at least one homozygous mutation or two heterozygous mutations. Heterozygous mutations have been confirmed on the two alleles by segregation analysis (Table 1).

All participants underwent a complete ophthalmologic examination including Best Corrected Visual Acuity (BCVA), intraocular pressure, axial length, automated visual field, and EDI OCT.

Demographic data and medical history (including the age of diagnosis of Usher Syndrome, the first symptoms onset, and the presence/absence of other members of the family affected by the same disease) were collected using a questionnaire that all the patients were asked to complete before the inclusion in the study. Duration of disease was considered from the date of first diagnosis.

Factors that have been demonstrated to influence choroidal thickness (refractive error > 6 diopters and elongated axial length) or poor OCT scan quality (media opacities, nystagmus) were considered noninclusion criteria. Other exclusions included history of glaucoma or uveitis, prior ocular surgery (excepted cataract surgery), systemic cardiovascular disease like hypertension, and diabetes.

We arbitrarily considered 3 stages of disease according to the parameters of visual acuity and visual field: stage 1 for patients with visual acuity better than 0.6 decimal or visual field mean defect less than 10 dB, stage 2 for patients with visual acuity from 0.6 to 0.2 decimal or visual field mean defect from 10 to 20 dB, and stage 3 for patients with visual acuity less than 0.2 decimal or visual field mean defect more than 20 dB.

### 2.1. Automated Visual Field

Visual field analysis was obtained using the 30-2 SITA standard program of the Humphrey Visual Field Analyzer (Zeiss/Humphrey Systems, Dublin, CA, United States).

Examinations were performed using the best correction for near vision. For the purpose of the study mean defect (MD) and Pattern Standard Deviation (PSD) values were considered.

### 2.2. Axial Length

Axial length was measured using the IOL Master 500 biometer (Carl Zeiss Meditec, Dublin, CA, United States). The values used for the study were obtained from the mean of five consecutive measures.

### 2.3. Enhanced Depth Imaging Optical Coherence Tomography (EDI OCT)

Retinal and choroidal imaging were obtained using Spectralis HRA and OCT (Heidelberg Engineering, Heidelberg, Germany). EDI OCT technique, previously described by Spaide and Margolis [5, 6], allows detailed imaging of the choroidal layer. For the study we took single line scans of 30° composed of 100 averaged images using the automatic eye tracking software across the fovea both vertically and horizontally.

**Figure 1:**
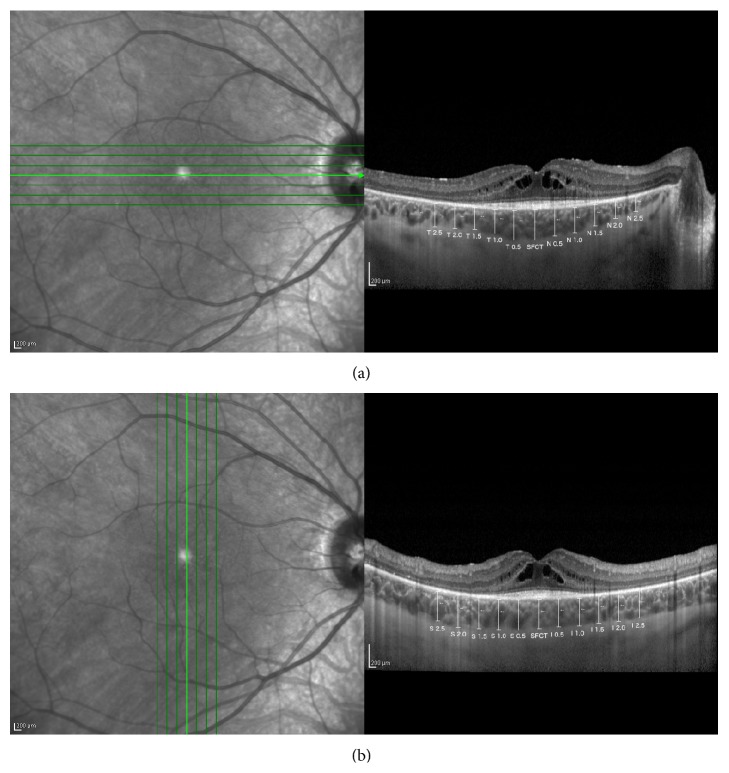
Horizontal (a) and vertical (b) choroidal and retinal measurements were taken manually in a masked fashion by two experienced OCT-readers. Values in the black boxes represent the topographic location of each measurement (T = temporal, N = nasal, S = superior, I = inferior, and SFCT = subfoveal choroidal thickness).

Both retinal and choroidal measures were taken manually in a masked fashion by two experienced OCT-readers as shown in Figure 1. Choroidal thickness was measured as previously described by Dhoot et al. [7]: from the inner border of the sclera to the outer border of the RPE vertically using the calipers of the Heidelberg reader software subfoveally and at 500 mm intervals for 2.5 mm nasal, temporal, superior, and inferior to the centre of the fovea. Retinal thickness was taken in the same intervals vertically from the outer border of the RPE to the inner border of ILM. Horizontal spatial distribution of photoreceptor layer was manually measured using the calipers. Also presence/absence of cystoid macular edema was considered.

All scans were performed with dilated pupils (using 1% tropicamide).

Only OCT scans of good quality were used for the measures (Figure 1).

### 2.4. Statistics

Data were analyzed using a Mixed Effect Model (MEM) approach. First, we studied the correlation between choroidal thickness and retinal thickness in the foveal region with a series of predictors (age, time from first symptoms, axial length, BCVA, MD, and PSD as continuous variables, presence of photoreceptors, and presence of oedema as categorical variables). Correlation between choroidal thickness and retinal thickness was also calculated. All correlations were calculated using univariate MEM models where a patient random effect was included to account for the covariance among observations from the same subject (i.e., two measurements from each patient, one for each eye). Secondly, we analyzed the effect of the age of the patients on the choroidal thickness at various distances from the fovea (every 0.5 microns from −2.5 to 2.5 microns away) on both temporal-nasal and superior-inferior axes. In this case a multivariate MEM analysis was used. Age and the site of measurement were treated as fixed effects; a patient and an eye random effect were used to correct for the covariance among observations from the same patient and the same eye (i.e., multiple measurements from each eye of each patient). Finally, an interaction term was included to allow different slopes in the model at each site. The different slopes were used to create a correlation map showing the effect of the age of the patient on the choroidal thickness at each site.

*Z*-test analysis was used to compare our data on choroidal thickness in patients with Usher Syndrome type 2 with values of choroidal thickness in healthy and nonsyndromic RP patients as reported in published literature.

## 3. Results

10 patients (20 eyes) with clinical and genetic diagnosis of Usher Syndrome type 2 were included in the study (4 men and 6 women). The mean age was 43.1 ± 13.09 years; the mean duration of disease was 17.8 ± 12.28 years.

Mean BCVA was 0.66 decimal, ranging between 0.05 and 1. Spherical equivalent ranged from −3.5 D to +2.5 D and mean axial length was 23.59 ± 1.29 mm (Table 2).

Mean subfoveal choroidal thickness (SFCT) (Table 3) was 237.45 ± 76.81 microns, with a mean choroidal thickness thinner 2.5 mm nasally than 2.5 mm temporally to the fovea (130.2 and 194.6 mm, resp.) and 2.5 mm inferiorly than 2.5 mm superiorly to the fovea (200.1 and 219.75 mm, resp.).

Mean foveal retinal thickness was 214.75 ± 75.72 microns. The mean retinal thickness was thinner 2.5 mm temporally than 2.5 mm nasally to the fovea (199.9 and 223.85 mm, resp.) and 2.5 mm inferiorly than 2.5 mm superiorly to the fovea (209.35 and 222.55 mm, resp.).

25% of the eyes (5/20) presented CME. In 15% of the eyes (3/20) photoreceptors outer segment layer was not detectable at OCT. When detectable, the mean horizontal extension of photoreceptor layer (concentric to the fovea) was 264.7 ± 148.71 microns.

SFCT was significantly correlated with age: SFCT decreases with age (estimated coefficient −4.412, Pearson correlation coefficient −0.725, *p* < 0.01). No statistically significant correlation was found between SFCT and duration of disease, different stages of disease (as evaluated with visual acuity and visual field parameters), retinal thickness, and axial length (Table 4).

**Figure 2:**
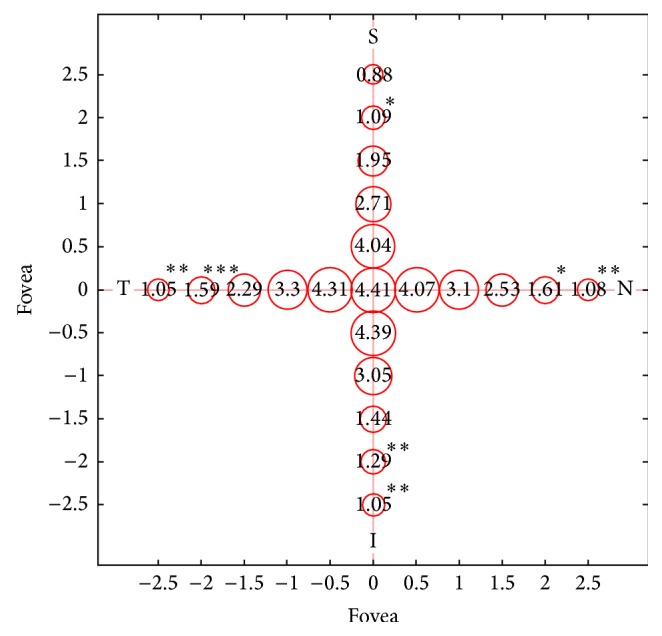
Correlation between the age of the patient and the choroidal thickness at different sites. Circle radii are proportional to the estimated correlation. Numbers represent the beta coefficients of the multivariate model for age at different sites (calculated from the interaction term in the model). Asterisks indicate in which sites the correlation between choroidal thickness and age is significantly different from the one found in the subfoveal region (chosen as the reference level in the model).

We then analyzed the correlation between choroidal thickness and age in each interval: Figure 2 shows the intervals in which the correlation was significantly different from that of the fovea. The intervals further away from the fovea were less correlated with age (the values indicated with the asterisks).

Choroidal thickness values from Usher patients were compared with values reported in published literature for healthy subjects and nonsyndromic RP patients. Choroidal thickness in Usher patients was significantly reduced when compared to healthy subjects [6] (*p* < 0.01, *Z*-test), while no difference was found when compared to choroidal thickness from nonsyndromic RP patients [8] (*p* = 0.72).

## 4. Discussion

Our case control study showed that choroidal thickness is reduced in people with Usher Syndrome type 2 if compared to healthy subjects [5, 6, 9, 10].

Our results confirmed those reported by Dhoot et al. [7], who studied choroidal thickness in nonsyndromic RP using the same strategy to analyze OCT images, by Ayton et al. [8] who studied nonsyndromic RP patients using a graphic software to analyze choroidal thickness in OCT scans and by Yoon and Yu [11] who used a different instrument of acquisition and analysis of OCT scans.

Both retinal vascularization and choroidal vascularization were reported to be significantly reduced in RP patients and in animal models of RP [1, 3, 4]: our observations can be considered a sign of this blood flow reduction.

Correlation between choroidal thickness and course of the disease remains unclear. Recent studies demonstrated different visual acuity, visual field, and cone electroretinogram amplitude loss in Usher Syndrome patients if compared to nonsyndromic type of RP [12]: comparison between our data and choroidal thickness values in nonsyndromic RP reported in literature could not explain these differences.

Although previous studies reported that choroidal arteries and choriocapillaris under macular region fill more rapidly and intensely compared to other retinal locations, supporting the concept that thicker choroidal thickness under the fovea is due to macular higher metabolic request [13]; however, similarly to Yeoh et al. [14] and Dhoot et al. [7], our study did not show any correlation between SFCT and visual acuity. We also could not find any correlation between SFCT and presence/absence of outer retinal layer or macular edema. A possible explanation of these data could be that EDI OCT technique does not allow distinguishing choriocapillaris, which represents about 10% of all choroidal thickness [15, 16], from the rest of choroidal volume. We are not able to understand which role choriocapillaris plays in this degenerative process.

First publications by Spaide and Margolis on choroidal thickness in healthy subjects reported a decrease of choroidal thickness with age [5, 6]. Similarly our study found a statistically significant correlation: also in patients with Usher Syndrome type 2 SFCT decreases with age. On the other hand, no correlation between SFCT and duration of disease was found, in contrast with Ayton's previous report [8]. This observation could be due to the fact that it is difficult to establish the exact onset of the disease: for the majority of patients diagnosis follows symptoms onset and in addition to that considering that Usher Syndrome is a genetic disease the concept of duration of disease is not as relevant as chronological age.

Interestingly in our Usher Syndrome study group the topographic choroidal thickness variation appears to be similar to topographic choroidal thickness variation previously reported in literature for healthy subject [17]. Choroidal thickness was thinner in nasal than in temporal sector and in inferior than superior sector. Furthermore we evaluated the correlation between choroidal thickness and age in each topographic sector: as shown in Figure 2 intervals far from the fovea resulted in being statistically less associated with age if compared to the fovea. This fact could be reasonably explained by the fact that central retina, particularly the fovea, is the last involved with the degenerative process in rod-cone dystrophy. To our knowledge, the relationship between the thickness of the choroid in different regions of the macula and the correlation with topographic variations has not yet been described.

Lack of correlation between choroidal thickness and axial length could be explained by the small number of patients studied and by the fact that we considered an exclusion criteria refractive error > 6 diopters.

In conclusion we have demonstrated in vivo choroidal thickness reduction in different topographic sectors in patients with Usher Syndrome type 2. These data are important not only for the comprehension of pathogenetic mechanisms in hereditary retinal diseases but also for the evaluation of therapeutic approaches. A deep knowledge of residual choroidal blood flow and changes in choroidal vascularization would be necessary for both retinal implant and stems cells therapy.

Limits of our study are represented by the small number of patients and the absence of a healthy and a nonsyndromic RP control group.

Further studies will need to investigate the correlation between different type of RP, classified by genetic mutation, and choroidal changes. New and more sophisticated OCT techniques will allow better understanding of the role that the choroid plays in the pathogenesis of hereditary retinal diseases.

## Figures and Tables

**Table 1:** USH2A gene mutations in our Usher patients cohort.

Patient	Mutation		Genotype
1	c.10450C>T	c.2276G>T	Heterozygous
2	c.5776+1G>C		Homozygous
3	c.1663C>G	c.2276G>T	Heterozygous
4	c.2276G>T	c.1663C>G	Heterozygous
5	c.1434G>C	c.4106C>T	Heterozygous
6	c.2898delG		Homozygous
7	c.11864G>A	c.4714C>T	Heterozygous
8	c.2299delG	c.1606T>C	Heterozygous
9	c.1751G>T	c.2276G>T	Heterozygous
10	c.2299delG	c.14074G>A	Heterozygous

**Table 2:** Demographic data.

Demographic data	Mean	Standard deviation
Age (years)	43.10	±14.09
Duration of disease (years)	17.80	±12.28
Visual acuity (decimal)	0.66	±0.37
Spherical equivalent (diopters)	−3.50	±2.50
Axial length (mm)	23.59	±1.29

**Table 3:** Mean choroidal thickness values at different sites (values are in microns).

Choroidal thickness	Mean	Standard deviation
Subfoveal	237.45	76.81
0.5 mm nasal	230.85	88.85
1.0 mm nasal	212.40	81.47
1.5 mm nasal	176.70	67.74
2.0 mm nasal	151.00	62.79
2.5 mm nasal	130.20	65.34
0.5 mm temporal	242.75	76.53
1.0 mm temporal	231.90	70.39
1.5 mm temporal	223.30	62.34
2.0 mm temporal	205.65	59.53
2.5 mm temporal	194.60	63.41
0.5 mm superior	233.40	71.61
1.0 mm superior	227.60	71.63
1.5 mm superior	220.90	74.97
2.0 mm superior	219.50	57.82
2.5 mm superior	219.75	64.19
0.5 mm inferior	228.40	75.70
1.0 mm inferior	222.20	74.19
1.5 mm inferior	209.45	71.62
2.0 mm inferior	202.95	64.94
2.5 mm inferior	200.10	67.79

**Table 4:** Beta regression coefficients of subfoveal choroidal thickness on different predictors (univariate analysis). Each column corresponds to a different regression analysis showing the estimated regression coefficient and, in brackets, the corresponding standard error. Asterisks represent the significance according to the legend on the bottom. Among tested variables, age is the only significant predictor of the subfoveal choroidal thickness.

	Dependent variable: subfoveal choroidal thickness								
	Retinal thickness	Age	Duration of disease	Axial length	BCVA	MD	PSD	Photoreceptors	Edema
Coefficients	0.093	−4.412^∗∗∗^	−1.475	0.046	28.314	1.585	13.075	−41.027	−8.168
	(0.216)	(1.370)	(2.151)	(19.061)	(68.411)	(3.608)	(8.391)	(35.597)	(34.146)

Intercept	228.185^∗∗∗^	437.738^∗∗∗^	273.859^∗∗∗^	246.514	228.984^∗∗∗^	288.786^∗∗∗^	157.069^∗∗^	253.754^∗∗∗^	249.642^∗∗∗^
	(51.328)	(61.449)	(45.756)	(450.333)	(51.608)	(97.277)	(64.992)	(23.881)	(26.223)

Observations	20	20	20	20	20	20	20	20	20

*Note*.; ^∗∗^ *p* < 0.05; ^∗∗∗^ *p* < 0.01.
